# PoPoolation: A Toolbox for Population Genetic Analysis of Next Generation Sequencing Data from Pooled Individuals

**DOI:** 10.1371/journal.pone.0015925

**Published:** 2011-01-06

**Authors:** Robert Kofler, Pablo Orozco-terWengel, Nicola De Maio, Ram Vinay Pandey, Viola Nolte, Andreas Futschik, Carolin Kosiol, Christian Schlötterer

**Affiliations:** 1 Institute of Population Genetics, Vetmeduni Vienna, Vienna, Austria; 2 Department of Statistics, University of Vienna, Vienna, Austria; Erasmus University Medical Center, Netherlands

## Abstract

Recent statistical analyses suggest that sequencing of pooled samples provides a cost effective approach to determine genome-wide population genetic parameters. Here we introduce PoPoolation, a toolbox specifically designed for the population genetic analysis of sequence data from pooled individuals. PoPoolation calculates estimates of *θ*_Watterson_, *θ*_π_, and Tajima's D that account for the bias introduced by pooling and sequencing errors, as well as divergence between species. Results of genome-wide analyses can be graphically displayed in a sliding window plot. PoPoolation is written in Perl and R and it builds on commonly used data formats. Its source code can be downloaded from http://code.google.com/p/popoolation/. Furthermore, we evaluate the influence of mapping algorithms, sequencing errors, and read coverage on the accuracy of population genetic parameter estimates from pooled data.

## Introduction

The recent advances in sequencing technology have changed our experimental approaches to biological questions. It has become possible to move from small scale, gene centric studies to genome-wide analyses and remain within the budget of individual research grants. Even population genetic analyses have become within the reach of moderate research budgets by sequencing pools of individuals [e.g.: 1], [2]

The new sequencing technologies have also changed the time allocation within a research project as well as the training required. Classic population studies typically involved a considerable wet-lab component for data collection. The new sequencing technologies reduce wet-lab work to DNA extraction and library construction. The analysis of the massive amounts of data generated in the course of a single experiment not only requires more time, but also new skills.

The challenges of Next Generation Sequencing data, namely a hitherto unprecedented number of extremely short sequence reads containing more sequencing errors than previous sequencing technologies, have lead to the development of many new software tools over the past few years. For many applications, such as SNP (single nucleotide polymorphism) discovery [e.g.: 3], [4], [5], RNA-Seq [e.g.: 6], [7], ChIP-Seq [e.g.: 8], [9], and de novo assembly [e.g.: 10], [11], users can choose among a variety of software tools either in the public domain or from commercial software suppliers. For population genetic analyses, software tools are targeted at the analysis of individual genome sequencing projects [e.g.: 12]. To our knowledge no software packages are publicly available for population genetic analysis of pooled sequence data.

Here, we introduce PoPoolation, a software suite specifically tailored for the analysis of pooled samples for population genetic inference. Furthermore, we carefully evaluate how peculiarities of the Next Generation Sequencing data (such as sequencing errors, mapping to a reference genome and read coverage) affect population genetic inferences.

## Results

**Figure 1:**
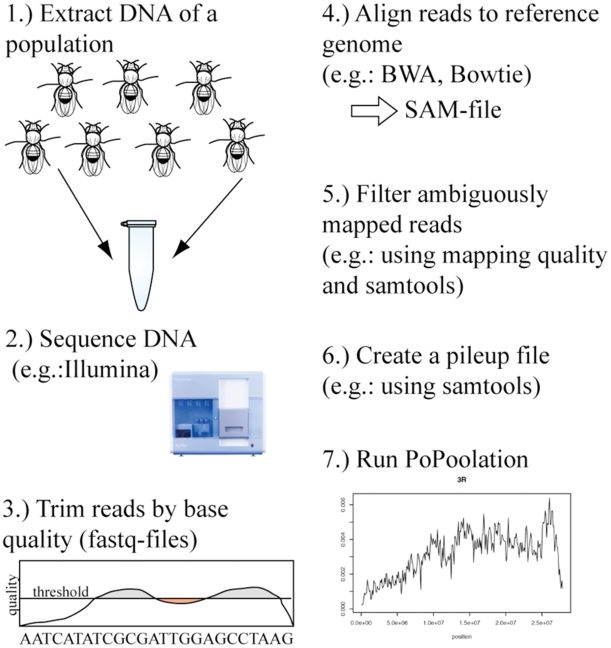
Outline of a population genetic analysis from pooled sequence data. Sequencer figure from http://www.illumina.com/

The analysis of short sequence reads from pooled DNA samples requires several steps, as indicated in Figure 1. The first step in processing the data is trimming of the reads. Table 1 shows how trimming parameters influence the average length and quality of the reads used for mapping. While only few reads are lost with a quality threshold of 10 or 20, almost 70% are lost when a quality of 30 is used. We found that a threshold of 20 with a minimum length of 40–50 bp reliably generates high quality data. After trimming the reads for low quality bases, reads are mapped against a reference genome with the Burrows-Wheeler Alignment Tool (bwa [15]). Using SAMtools [15] the aligned reads are converted into a pileup file. This pileup file is used by PoPoolation to perform population genetic analyses.

**Table 1:** Trimming statistics of 14×10^6^ reads.

	No trimming	0*	10	20	30
% reads passing trimming	100	99.73	91.93	88.92	33.49
Sum read length [Mbp]	1081.22	1077.42	960.57	912.08	298.65
Average read length	76.00	75.94	73.45	72.10	62.68
Average quality	27.50	27.56	29.51	29.90	32.23

0*: trimming includes removal of ‘N’-characters at the end of reads.

### Validation

**Figure 2:**
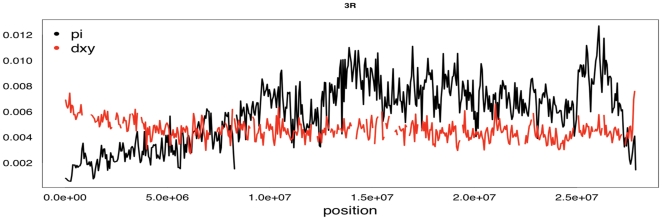
Graphical output of polymorphism and divergence estimates using PoPoolation. Sliding window analysis of *θ*_π_ of a Portuguese *D. melanogaster* population on chromosome 3R (black line). The red line shows divergence (dxy) between *D. melanogaster* and *D. simulans* using the same window size and step size as for *θ*_π._ Note that dxy is scaled by 1/10. Both lines are based on non-overlapping windows of 50 kb.

Figure 2 shows the polymorphism and divergence pattern along the 3R chromosome of *D. melanogaster*. Our analysis captures important features of variability in *D. melanogaster*: regions close to the centromere and telomere (located at the left and right ends of figure 2, respectively) show the well-described drop in variability.

Another striking feature apparent in Figure 2 is the ragged pattern of polymorphism, which shows 2-fold differences in variation between some regions in close physical proximity. To validate that this pattern reflects heterogeneity in sequence variation rather than problems with our pooling approach, we compared the polymorphism pattern on 3R obtained from a Portuguese *D. melanogaster* population to the polymorphism data generated by Sanger re-sequencing of a *D. melanogaster* population from The Netherlands [21]. Using the targeted regions option of PoPoolation (see below) we found a high correlation between our variability estimates and the ones published by Hutter et al. (2007) for the Dutch population (*θ*_Watterson_ ρ = 0.78, *p-value*<2.2×10^−16^ and *θ*_π_ ρ = 0.82, *p-value*<2.2×10^−16^; Table S1). Nevertheless, we also noted that the average variability in the Portuguese population was higher than for the Dutch population (*θ*_Watterson_: 0.0084 vs. 0.0065, Wilcoxon sum rank test *p-value*: 2.118*10^−6^; *θ*_π_: 0.0075 vs. 0.0063, Wilcoxon sum rank test *p-value*: 0.004567).

Three important sources of error could affect the population genetic analysis of pooled samples: sequencing errors, problems with mapping the reads to the reference and insufficient sequence coverage. In the following, we evaluate all three factors.

### Sequencing errors

The typical error rate of unprocessed reads from an Illumina sequencer is about 1%. As sequencing errors inevitably affect the polymorphism estimates and Tajima's D, it is highly desirable to reduce the sequencing error. It has been proposed to condition on a minor allele count larger than one, resulting in a truncated allele frequency spectrum [22]. We performed computer simulations to evaluate whether this correction is sufficient. We simulated 400 chromosomes (100 kb each) with the ms software [20]. The simulated chromosomes were re-coded into DNA sequence data by using a *D. melanogaster* chromosome as template. Finally, we generated random reads from these chromosomes with a sequencing error of 0.1–1%. These reads were then fed into the analysis pipeline of PoPoolation and the variability estimators were calculated. Our simulations show that with low error rates of 0.1–0.2% a minor allele count of two is well suited for a coverage up to 100, while for higher coverage a minor allele count of three is needed. Nevertheless, for an error rate of 1%, even a minor allele count of three is insufficient.

Alternatively, it is possible to reduce the sequencing error by incorporating adequate quality control measures such that even low frequency alleles could be reliably detected and quantified [23], [24]. We evaluated whether simple quality measures could lead to a sufficient reduction in the error rates of Illumina reads to make their use in pooling experiments feasible. We determined the influence of trimming on the error rate of 74 bp (base pairs) Illumina sequence reads using the PhiX control lane of a GAIIx with sequencing chemistry v 3. The error rate of unprocessed reads was about 1%. After trimming the PhiX reads with a quality cutoff of 20 the error rate was reduced by an order of magnitude to 0.15%. Further reductions in error rate were achieved by conditioning on a minimum sequence quality of every SNP (e.g.: 0.07% for a sequence quality of 20). Hence, simple quality control measures that do not discard a large fraction of the sequence reads (Table 1) are sufficient to reduce the sequencing error to an extent that reliable population genetic analyses of pooled samples are possible with a minor allele count of two or three.

**Figure 3:**
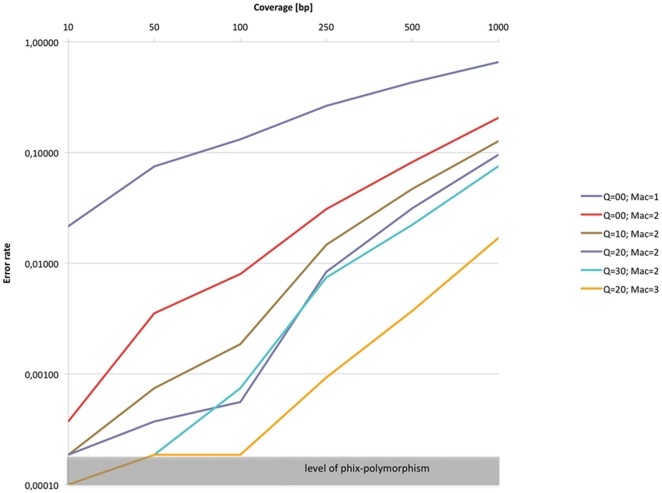
Sequencing errors in relation to coverage, minor allele count, and sequence quality. PhiX sequences (74 bp) generated with an Illumina GAIIx sequencer were analyzed for sequencing error rate (number of mutated bases after quality filtering). The gray bar indicates the presence of a polymorphic site in the PhiX sequence, which results in a minimum sequencing error rate.

In our computer simulations we assumed that all sequencing errors are independent. If sequencing errors are biased, the same error may be generated more frequently than assumed, leading to an inflated variability estimate. As this effect is difficult to simulate without knowing the exact bias, we decided to obtain an empirical error rate of pooled samples after using the quality control measures mentioned above: trimmed reads, sequencing quality, and minor allele count. We determined the efficiency of these measures by inferring the error rate (fraction of bases carrying at least one incorrectly identified SNP) in a pooling setting with different coverage. Please note that the definition of the error rate differs from the one used above to quantify the influence of trimming. Figure 3 shows that without quality filtering and with a minor allele count of one, a very low read coverage results in an extremely high error rate. Introducing a minor allele count cutoff has a profound effect on the error rate. By conditioning on a minimum of two counts the error rate is reduced by at least one order of magnitude. Filtering for quality further reduces the error rate by a factor of about five. For coverage between 100 and 200 it is advised to increase the minor allele count to three. An even higher coverage needs a further minor allele count increase. These analyses demonstrate that after accounting for sequence quality and choosing an adequate minor allele count, the effective number of sequencing errors is low enough to allow for reliable polymorphism analysis in sequence pools.

### Mapping errors

Mapping of sequence reads from pooled data is a challenging task. The population pool may contain alleles with a different number of substitutions relative to the reference genome. Hence, if the mapping parameters are too stringent some of the reads may not be mapped. Contrary to the sequencing of a single genome, these unmapped reads may remain unnoticed, in particular if the highly diverged reads occur at a low frequency. Overly liberal mapping parameters, on the other hand, increase the chance that a read is incorrectly mapped. Given the central importance of mapping, we evaluated different mapping strategies and show that most of them introduce a systematic bias and subsequently are not well-suited for a population genetic analysis.

While it is possible to test alignments with simulated reads, this strategy is restricted by the assumption that the simulations capture the pattern of variability observed in real sequences. The simulations may, for example, assume that polymorphisms are evenly distributed over the sequence and thereby ignore the fact that different parts of the genome have variable selective constraints. Hence, we did not rely on simulated reads to evaluate mapping parameters. Rather, we took advantage of paired-end reads from real pooling data and evaluated two aspects of mapping: 1) biased allele frequency estimates due to sequence divergence between reference and mapped reads and 2) mapping quality, i.e. incorrectly or unmapped reads.

**Figure 4:**
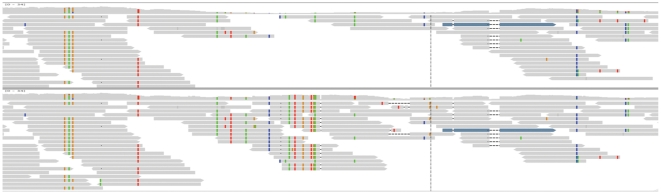
Improvement of the alignment for diverged regions using the PE-SW remap algorithm. IGV screenshot of the mapping of pooled sequence reads in a highly divergent region of *D. melanogaster*. The upper panel shows an alignment of the PE reads without the PE-SW remap and the lower panel shows the same region with the PE-SW remap.

Allele frequency bias: In comparison to mapping with global alignment, the frequency of the reference allele was on average 3% higher when a local alignment was used. The bias towards the reference allele with local alignments has been described before [25] and results from soft masking (i.e.: ignoring) the end of the read if a mismatch between reference and read is observed. Global alignments, however, aim to map the entire read. While this reduces the bias compared to local alignment mapping, some bias remains as highly diverged reads may not map at all. Hence, we also evaluated a mapping strategy which takes advantage of paired-end sequencing: the two reads of a pair are mapped individually using global alignment without a seed, and if only one of the two reads is mapped, the other one is aligned by local alignment. We will refer to this strategy as PE-SW-remap throughout the manuscript. Two thirds of the reads showed no difference and about 20% displayed a strong bias against the reference allele, suggesting a high frequency of highly diverged alleles. Figure 4 provides such an example where PE-SW-remap allows mapping of several reads carrying multiple non-reference alleles.

Mapping accuracy can be assessed via the number of correctly mapped paired-end reads. Improper spacing between the paired-end reads, mapping to two different chromosomes, mapping in the wrong direction and unmapped mates are indications of problems with mapping. As expected, the worst result was obtained when reads were mapped without allowing for gaps (Table 2). We also noted that the use of a seed for mapping resulted in fewer mapped reads and more broken pairs. Allowing for a higher sequence divergence improved the mapping (i.e.: fewer broken pairs). PE-SW-remap obtained the best mapping results using a global alignment without seeds. Figure 4 gives an example on how PE-SW-remap could improve the alignment.

**Table 2:** Comparison of mapping strategies.

seed	No	No	No	No	Yes	No
Max. # gap openings	0	0	1	2	1	2
Max. # mismatches	5	4	5	5	5	5
PE-SW-remap	No	No	No	No	No	Yes
Mapped	94.83	93.78	97.79	97.59	96.45	99.25
Proper pair	89.66	87.81	95.10	94.75	92.57	98.28
Mate not mapped	4.30	5.16	1.71	1.90	2.94	0.24
Mate mapped to wrong chromosome	0.53	0.50	0.59	0.58	0.57	0.46

30×10^6^ paired-end reads were used for mapping. Long insertions and deletions with 12 bp were allowed for mapping strategies including gaps. If seeding was used, the seed length was 32 bp.

### Stochastic errors

**Figure 5:**
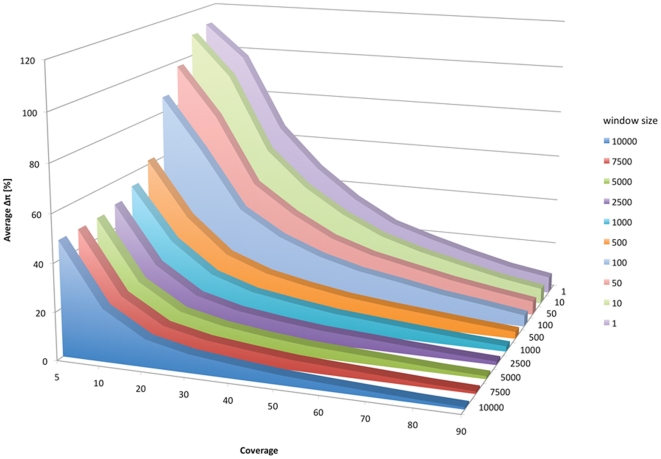
The influence of coverage and window size on the accuracy of the estimated *θ*_π_. The accuracy was measured as the mean standardized difference between *θ*_π_ estimated for a given window size and its expectation.

The accuracy of allele frequency estimates by sequencing of pooled individuals is highly dependent on the sequence coverage. If sequence coverage is low, it is better to obtain population estimators in a larger window to avoid incorrect estimates caused by stochastic error. PoPoolation provides the option to measure *θ*_Watterson_, *θ*_π_ and Tajima's D in a sliding window analysis with a variable window size. To avoid an arbitrary window size choice and provide some analytical guidelines, we determined the joint effect of window size and coverage on the accuracy of *θ*_π_. As expected, low coverage and small window sizes had a higher uncertainty (Figure 5). Nevertheless, 40-fold coverage in a 1 kb window produced highly reliable estimates, which suggests that this level of coverage is sufficient for a comparison of polymorphism among genes. Analyses requiring a reliable estimate for every SNP (which corresponds to SNP heterozygosity) require a much higher coverage. Even with 90-fold coverage, which was the highest level considered by us, we noted a considerable error.

## Discussion

Previous analyses showed that sequencing pooled DNA samples is a cost-effective approach to obtain genome-wide polymorphism data [22]. Here, we introduced PoPoolation, a software tool allowing a genome-wide polymorphism analysis using Next Generation Sequence reads. PoPoolation provides several options to retrieve polymorphism data for specific genomic regions of interest and allows this data to be displayed in FlyBase and the UCSC Genome Browser, thus linking polymorphism data with functional information.

Our analysis of the 3R chromosome arm showed that the inferred distribution of polymorphism along the chromosome closely fits that inferred by sequencing short PCR products distributed along the chromosome in a small number of flies (≤12) from The Netherlands [21]. Shallow sequencing of several individuals from a North American and an African population with 454 reads also resulted in a high correlation of the polymorphism estimates in comparisons with Dutch or Zimbabwean populations [26]. Interestingly, the 454 data showed less polymorphism than the data from Hutter et al. (2007), while the pooling data in our study were more polymorphic than the corresponding loci from the Dutch population. Furthermore, *θ*_Watterson_ was higher than *θ*_π_ in our data set. Interestingly, increasing the minor allele count up to five did not change the overall pattern, the Portuguese population remained more variable than the Dutch population and *θ*_Watterson_ was still higher than *θ*_π_ (data not shown). These results clearly demonstrate that the strong fluctuations in variability along the chromosome reflect a biological feature that is conserved across populations, rather than an artifact of pooling. As the Portuguese population has not been studied before, it is possible that its higher variability reflects a true biological property of this population, but we cannot exclude the possibility that it is an artifact of the mapping. Furthermore, our data also shows how the pattern of variation in the 3R chromosome decreases towards the centromere and telomere. As the divergence between *D. melanogaster* and *D. simulans* does not follow this pattern, the drop in variability cannot be explained by mutation rate variation, but is attributed to selection [27], [28].

As a high sequencing error rate as well as erroneous mapping of reads could have inflated the variability estimate, we very carefully evaluated the sequencing error rate and mapping accuracy. Our results indicate that very simple quality control measures, such as trimming of reads and conditioning on a moderate sequence quality of 20 reduces the sequencing error by more than one order of magnitude to about 0.01%. The computer simulations indicated that for this sequencing error rate it is sufficient to condition on a minor allele count of two or three to obtain population genetic summary statistics that are close to the expectation. While all these results suggest that the variability estimators obtained in our pooling study have been correctly inferred, we cannot rule out that some sequencing and mapping errors affected our estimates. Nevertheless, the high correlation in variability estimates between sequencing of pooled individuals and Sanger sequencing indicates that the analysis of pooled samples correctly recovers heterogeneity in variability patterns across the genome. Hence, we anticipate that the analysis of pooled samples will become highly popular for the comparison of polymorphism patterns along the genome and between populations. Furthermore, experimental evolution studies will greatly benefit from sequencing pooled DNA samples to identify the spread of beneficial mutations in an outcrossing population. With PoPoolation we have provided a tool that allows users with limited bioinformatic skills to take advantage of Next Generation Sequencing of pooled DNA samples and to obtain genome-wide polymorphism patterns. We expect in the near future to also incorporate other statistics of interest for population genomics, like the McDonal-Kreitman test [29], the HKA test [30] and tests for synonymous vs. non-synonymous polymorphisms.

## Materials and Methods

### Fly samples

113 isofemale lines of *D. melanogaster* were collected 2008 in Northern Portugal (Povoa de Varzim). The isofemale lines were kept in the laboratory for five generations and five females from every line were combined into a pool of flies for sequencing.

### Sequencing

Female flies were homogenized and DNA was extracted with the Qiagen DNeasy Blood and Tissue Kit (Qiagen, Hilden, Germany). We used the Genomic DNA Sample Preparation Kit (Illumina, San Diego, CA) to generate paired-end libraries. Five µg DNA were sheared with a nebulizer, and after end repair, A-tailing and ligation of paired-end adapters the library was size-selected on an agarose gel (300 bp) and amplified using 10 PCR cycles.

Cluster amplification was performed using a Paired-End Cluster Generation Kit v2. Sequences were generated with the Illumina Sequencing Kits v3 on a Genome Analyzer IIx.

Image analysis was performed with the Firecrest, Bustard and Gerald modules of the Illumina pipeline v. 1.4.

### Mapping of reads

For all analyses presented in this manuscript we used bwa [13] to map reads against the *D. melanogaster* (version 5.18) reference genome. Nevertheless, it is important to note that PoPoolation is based on the widely used SAM format allowing for the use of alternative mapping software provided that this software generates a SAM file.

### Trimming statistics and error rates

Sequencing errors are not evenly distributed along sequence reads [14]. The error rate increases with the position in the read. Additionally, the first base often has an elevated error rate. To account for this pattern of sequencing errors, we implemented a modified Mott algorithm from Phred (http://www.phrap.org/phredphrap/phred.html). This algorithm identifies the highest scoring substring of every read given a quality threshold and trims the read from either side until only bases of this substring are kept. In addition, the user can specify a minimum number of bases for each read to be kept in the data set.

We used one lane of PhiX reads (14×10^6^) with a length of 74 bp and an error rate of 1.03% as estimated by the Illumina pipeline 1.4.0. The reads were trimmed with the script trim-fastq.pl using different quality thresholds (0, 10, 20, 30) and a minimum length of 50. The trimming statistic was generated using a custom Perl script. To calculate the error rates, reads that were trimmed with a quality threshold of 20 were mapped to the PhiX genome, filtered for a mapping quality of 20 and converted into a pileup file. The error rate for a given quality threshold was calculated as the number of mismatches meeting the quality requirement divided by all bases meeting the quality requirement.

### Comparison of mapping algorithms

Several strategies can be pursued to map reads. The most simple and fastest strategy specifies a sequence string (seed) that needs to be mapped against the reference with a specified maximum number of mismatches. The mapped seed is then extended, either using local or global alignment. Local alignment does not attempt to match the full read. This inevitably leads to the omission of SNPs, particularly at the ends of the read, causing a bias towards the character state in the reference genome. The global mapping strategy avoids this bias, but requires an a priori specification of the maximum number of inserted/deleted bases and an upper bound for the number of substitutions in the read. The limitation of this approach is that the success of the mapping depends on the correct specification of these mapping parameters. Irrespective of whether local or global alignments are used, the seed restricts the divergence of the read to the reference genome. Hence, an alternative mapping strategy avoids the use of seeds at the expense of computational speed. The third mapping option takes advantage of paired-end reads. Both reads are initially mapped separately, and if one read of the pair cannot be mapped it is aligned using a local alignment procedure (Smith-Waterman). Throughout the manuscript, we refer to this mapping strategy as PE-SW-remap.

A single lane of *D. melanogaster* paired-end data was used (SRA023610.1). 36×10^6^ 74 bp reads were trimmed with the script trim-fastq.pl using a quality threshold of 20 and a minimum length of 40. A total of 15098991 (84%) paired-end reads met the requirements. These reads were mapped to the *D. melanogaster* genome (version 5.18) using ‘bwa aln’ [13] with the following parameters: seeding of the reads (-l), the allowed error rate (-n), the number of gap openings (-o), and the Smith-Waterman alignment of the unmapped mate with ‘bwa sampe’. If not mentioned otherwise the gap length was set to 12 (-e and -d). The maximum allowed distance between reads was set to 500 bp. We used single reads to evaluate the bias towards the reference allele with local and global alignments, as bwa does not support local alignment of paired reads. The choice of the exact mapping parameters is highly dependent on the polymorphism pattern and levels in the target organism. Hence, we focused on the comparison of different strategies (algorithms) to map short reads, as these results could be generalized.

### Estimating the number of false positive SNPs using PhiX

We used one lane of PhiX reads (14×10^6^) with a length of 74 bp and an error rate of 1.03% as estimated by the Illumina Pipeline 1.4.0. The reads were trimmed with the script trim-fastq.pl using a quality threshold of 20 and a minimum length of 40. All trimmed reads were mapped to the reference genome of PhiX using ‘bwa aln’ [13] with the parameters ‘-o 2 -e 12 -n 0.01 -l 100 -d 12’. We did not attempt to match pairs, thus this analysis rests effectively on single reads. The mapping results were filtered for a mapping quality of 20 and converted into a fastq file. We randomly sampled reads from the resulting fastq file to obtain PhiX coverages of 10, 50, 100, 250, 500 and 1000 with different quality thresholds of 0, 10, 20 and 30. The randomly sampled reads were again mapped to the PhiX reference using ‘bwa aln’ [13] with the parameters ‘-o 2 -e 12 -n 0.01 -l 100 -d 12’. The mapping results were converted to pileup files with SAMtools [15]. SNPs were called from the pileup files with a custom script using different minor allele counts of 1, 2, 3 and different quality thresholds of 0, 10, 20 and 30.

### Features implemented in PoPoolation

The widely used population genetics parameters *θ*_Watterson_ and 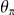 were designed for sequencing of individuals. We have implemented unbiased estimates for pooled samples with poolsize 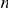 and coverage 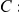


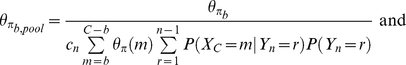


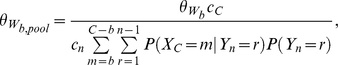
where 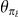 and 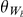 are modified versions of the classical 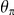 and 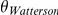 that are only evaluated on SNPs with minimum allele count of 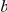.

Furthermore, 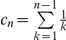, 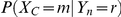 is the probability of having allele frequency 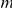 among the reads given an allele frequency 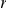 in the pool and 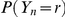 is the probability that an allele has frequency 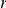 in the pool.

These two parameter estimators account for the truncated allele frequency spectrum (see below) and re-sequencing of the same chromosomes, as described in [14].

Tajima's D is a classic summary statistic characterizing deviations from the null model of a constant size population without selection [16]. PoPoolation uses a modified Tajima's D that accounts for the truncated allele frequency spectrum (see below) used for pooled data:
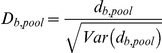
with
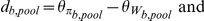


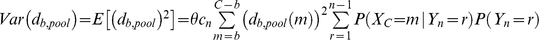
, where *θ* is estimated by 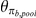 in the same window in which 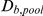 is calculated. Hereby we assume that all individuals contribute roughly equal amounts of DNA to the pool.

To facilitate the interpretation of genome-wide polymorphism data, PoPoolation also calculates sequence divergence for closely related species pairs. For this purpose complete genomes are aligned with the MAUVE alignment software [17] and sequence divergence is calculated.


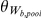
, 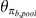, 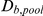 and divergence are calculated for a specified DNA fragment of interest or for all aligned fragments of the genome. To analyze heterogeneity between different chromosomal regions, a sliding window analysis is used. PoPoolation allows the user to specify the window size as well as step size.

PoPoolation generates a simple graphical overview of the polymorphism pattern along a chromosome (Figure 2). Furthermore, PoPoolation also produces a file in the wiggle format (http://genome.ucsc.edu/goldenPath/help/wiggle.html) that can be loaded by the Integrative Genomics Viewer (http://www.broadinstitute.org/igv/). Alternatively, the wiggle file can be uploaded on the UCSC Genome Browser [18] allowing the user to link population genetic analyses with the relevant genome annotation and other functional features. Because FlyBase [19] does not accept wiggle files, PoPoolation generates a special output file that enables the polymorphism pattern to be linked with FlyBase.

While the sequencing of pooled samples generates genome-wide polymorphism patterns, researchers are often only interested in one or a few genomic regions. PoPoolation provides the option to restrict the analysis to regions specified in a gene transfer format (GTF) input file (http://genome.ucsc.edu/FAQ/FAQformat.html). Apart from polymorphism and divergence data, PoPoolation also supplies a table of polymorphic sites for the specified region. Alternatively, researchers may be interested in excluding specific regions from the analysis. Repetitive sequences, for example, are notoriously difficult to handle with Next Generation Sequencing reads. Hence, it is possible to use a gtf file to mask genomic regions containing repetitive sequences. Similarly, genomic regions with known structural variants can be also excluded.

### Sensitivity of θ_π_ to window size and coverage

From a data set consisting of 212×10^6^ reads generated from the Portuguese population that were mapped to the *D. melanogaster* genome (version 5.18) with ‘bwa aln’ and the parameters ‘-o 2 -e 12 -n 0.01 -l 100 -d 12’, we extracted 40×10^6^ reads mapping to chromosome 3R (corresponding to ∼100-fold coverage). All reads with a minimum mapping quality of at least 20 were used as single read data and converted into a fastq file. From this fastq file, we randomly sampled reads to obtain a total coverage of 5, 10, 20, 30, 40, 50, 60, 70, 80 and 90 of chromosome 3R using a custom Perl script. The randomly sampled reads were mapped to chromosome 3R of *D. melanogaster* with ‘bwa aln’ and the parameters ‘-o 2 -e 12 -n 0.01 -l 100 -d 12’. Using a custom Perl script and the full data set, we identified 2000 SNPs (minor allele count = 4; minimum coverage = 8; minimum base quality = 20) on chromsome 3R, which are separated by at least 10,500 bp. Furthermore, we required that at least 90% of the 10,000 base pairs downstream of the SNP have a minimum coverage of 8 in the full data set. These high confidence SNPs were used to calculate *θ*_π_ for windows starting with the SNP. We calculated the difference in *θ*_π_ of the full data set to the respective values obtained using the reduced data set (for example coverage: 5, 10, 20 etc.) and standardized this difference by *θ*_π_ from the full data set. Note that this standardization accounts for the bias generated by conditioning each window to start with a SNP. The average over 2000 windows is reported in Figure 5. For SNPs other than the high confidence SNPs we required the following criteria: minor allele count >1, a minimum coverage of 4 and a minimum base quality of 20.

### Simulated reads

We used ms [20] to generate five datasets assuming a *θ* of 5×10^−3^, which matches *D. melanogaster* data (ms 400 1 -seed 1 17 666 -t 500 -r 2500 100000). *θ*_Watterson_, *θ*_π_, and Tajima's D were determined with the sample_stats software included in the ms package [20]. The output of ms was converted to DNA sequences using ms2dna (http://guanine.evolbio.mpg.de/cgi-bin/mlRho/mlRho.cgi.pl) with the 3R chromosome of *D. melanogaster* (position 10,000,0000 to 10,100,000) as template sequence. Short sequence reads were generated with Sequencer (http://guanine.evolbio.mpg.de/sequencer/) with assumed error rates 0.1%, 0.2% and 1%, and targeted coverage 50×, 100× and 250×. The simulated reads were mapped with bwa (-o 2 -e 12 -n 0.01 -l 100 -d 12) and processed with PoPoolation. We measured the relative difference between the estimates obtained from PoPoolation (*o*) and the expected (*e*) results calculated with sample_stats from the ms package [20] using the original ms output. For each set of parameter combinations, we repeated this procedure five times (*n*). The averages of the relative differences (
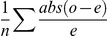
) are reported in Table 3 (*θ*_π_) and the Table S2 (*θ*_Watterson_ and Tajima's D).

**Table 3:** Effect of coverage and sequencing error rates on *θ*_π_.

		Cov 50	Cov 100	Cov 250
minor allele count 1	Error Rate 1%	3.93	3.94	3.94
	Error Rate 0.2%	0.82	0.83	0.83
	Error Rate 0.1%	0.41	0.42	0.42
minor allele count 2	Error Rate 1%	0.72	1.36	2.82
	Error Rate 0.2%	0.04	0.08	0.17
	Error Rate 0.1%	0.02	0.03	0.06
minor allele count 3	Error Rate 1%	0.093	0.25	1.12
	Error Rate 0.2%	0.01	0.02	0.03
	Error Rate 0.1%	0.01	0.01	0.02

Average relative mean absolute deviation between the observed and expected value of *θ*_π_. Expectations were obtained from ms (sample_stats) and compared to the observed value calculated with PoPoolation for three different coverage values and three different sequencing error rates. The observed *θ*_π_ was calculated assuming three different values of the minimum frequency of the alternative allele in the sequenced pool. Cov: Coverage.

## Supporting Information

Table S1 **Comparison of variability estimates for genomic fragments sequenced by traditional Sanger or by sequencing of pooled samples.**
(XLS)

Table S2 **Effect of coverage and sequencing error rates on Watterson's** ***θ*** **and Tajima's D.** Sequences were submitted to the short read archive [SRA023610.1].
(DOC)
