# Supplementary material for: PoPoolation: A Toolbox for Population Genetic Analysis of Next Generation Sequencing Data from Pooled Individuals
Source: PLoS One. 2011 Jan 6;6(1):e15925. doi: 10.1371/journal.pone.0015925 (PMC3017084; doi:10.1371/journal.pone.0015925)
Supplement: Table S2 — Effect of coverage and sequencing error rates on Watterson's θ and Tajima's D. Sequences were submitted to the short read archive [SRA023610.1]. (DOC) [file pone.0015925.s002.doc]

Table S2

Effect of coverage and sequencing error rates on Watterson’s *θ* and Tajima’s D.

| A) |  | Cov 50 | Cov 100 | Cov 250 |
| --- | --- | --- | --- | --- |
| MAC 1 | Error Rate 1% | 17.85976 | 25.95254 | 35.10063 |
|  | Error Rate 0.2% | 4.439792 | 7.625281 | 15.32105 |
|  | Error Rate 0.1% | 2.259066 | 4.024467 | 8.625291 |
| MAC 2 | Error Rate 1% | 2.066976 | 5.865316 | 19.32004 |
|  | Error Rate 0.2% | 0.097867 | 0.32521 | 1.568921 |
|  | Error Rate 0.1% | 0.03048 | 0.100519 | 0.473091 |
| MAC 3 | Error Rate 1% | 0.143503 | 0.756636 | 6.084897 |
|  | Error Rate 0.2% | 0.02689 | 0.025838 | 0.120205 |
|  | Error Rate 0.1% | 0.027385 | 0.014895 | 0.037693 |
| B) |  |  |  |  |
| MAC 1 | Error Rate 1% | -8.32538 | -16.9815 | -29.6343 |
|  | Error Rate 0.2% | -1.66467 | -5.34125 | -18.1832 |
|  | Error Rate 0.1% | -0.41473 | -2.46495 | -10.3128 |
| MAC 2 | Error Rate 1% | 0.100709 | -2.0037 | -12.3281 |
|  | Error Rate 0.2% | 0.027817 | 0.324178 | -0.53124 |
|  | Error Rate 0.1% | -0.03666 | 0.172797 | 0.249656 |
| MAC 3 | Error Rate 1% | -0.15665 | 0.379001 | -2.7012 |
|  | Error Rate 0.2% | -0.12033 | -0.02438 | 0.291085 |
|  | Error Rate 0.1% | -0.12127 | -0.03472 | 0.170824 |

Average relative mean absolute deviation between the observed and expected A) Watterson’s *θ* and B) Tajima’s D. Expectations were obtained from ms (sample_stats) and compared to the observed value calculated with PoPoolations for three different coverages and three different sequencing error rates. The observed statistics were calculated assuming three different values of the minimum frequency of the alternative allele in the sequenced pool. Cov: Coverage, MAC: minor allele count.
